# The effect of using sour cherry (*Prunus cerasus* L.) puree in tarhana formulations on nutritional value and functional properties of tarhana

**DOI:** 10.1002/fsn3.4191

**Published:** 2024-04-26

**Authors:** Ali Göncü

**Affiliations:** ^1^ Food Technology Program, Food Processing Department, Çine Vocational School Aydın Adnan Menderes University Aydın Turkey

**Keywords:** fermented, soup, sour cherry, tarhana, traditional

## Abstract

The aim of this study was to investigate the effects of using sour cherry puree in tarhana formulations on the nutritional value and quality criteria of tarhana. In the formulation, 50% and 100% of tomato and pepper purees were replaced by sour cherry puree, and in another formulation, 100% of sour cherry puree was used instead of yogurt. The use of sour cherry puree instead of tomato and pepper puree enriched the tarhana in terms of essential and other amino acids. Tarhana samples were found to be rich in oleic acid (22.02%–39.12%) among monounsaturated fatty acids. The presence of polyunsaturated fatty acids including linoleic (11.09%–33.56%) and linolenic acids (14.04%–28.91%) was determined in the samples. The dominant saturated fatty acid found in the samples was palmitic acid (8.05%–12.89%). There were no significant changes in moisture, *a*_w_, ash content, pH, HMF content as well as water and fat absorption capacity values. When sour cherry puree was used instead of yogurt, there was a decrease in protein (10.71%) and fat (0.38%) contents as well as *L* (43.46) value, while an increase was detected in total phenolic content (2.38 mg GAE/g), antioxidant activity (10.35 μmol TE/100 g) and mineral composition, namely K, Fe, Cu, Mn, and B. Considering sensory analyses, the sample in which tomato and pepper purees were substituted with 50% sour cherry puree received the highest scores in general taste, flavor, and consistency parameters together with the control tarhana (CT0). The 50% substitution of sour cherry puree with tomato and pepper purees enriched the tarhana samples both nutritionally and sensorially.

## Graphical abstract

The aim of this study is to investigate the effect of adding sour cherry puree to tarhana formulations on nutritional value and quality. The use of sour cherry puree has enriched tarhana in terms of essential amino acids and increased antioxidant activity. Additionally, it has been observed that sour cherry puree enhances the sensory properties of tarhana.

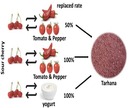

## 1 INTRODUCTION

Sour cherry (*Prunus cerasus* L.) is a stone fruit belonging to the *Rosaceae* family (Önem, 2017). The homeland of the sour cherry is probably a region lying between the Caspian Sea and the mountains of Northern Anatolia. It is also reported that wild sour cherries are found on Mount Olympus in Macedonia, in the mountainous parts of Italy and in central France (Önal, 2002). The top five sour cherry‐producing countries are Turkey, USA, Russia, Iran, and Ukraine (Kappel et al., 2012).

Sour cherry is one of the most attractive fruits in terms of nutritional value and sensory properties. It is characterized by its intense red color and sour–sweet flavor and contains many bioactive compounds beneficial to health. Organic acids that affect sour taste stimulate the secretion of digestive enzymes, vitamins, and minerals and are responsible for the smooth course of chemical reactions in the body (Nowicka et al., 2015). The potential health benefits of sour cherries have made them economically valuable. According to researchers in literature, sour cherries have been reported to be good for many diseases such as cardiovascular diseases, cancer, sleep disorders, and obesity (Kappel et al., 2012; Önem, 2017). This situation was attributed to the beneficial health effects of phyto‐compounds found in fruits. Sour cherry contains higher amounts of phyto‐compounds, especially phenolic substances, compared to many other fruits (Ferretti et al., 2010).

In sour cherries, the most common subgroups of polyphenols are procyanidins and anthocyanins, but colorless and pale yellow polyphenols are also found in significant amounts and have distinct biological activities. Polyphenols identified in sour cherry juices include anthocyanins as well as (−)‐epicatechin (flavanol), neochlorogenic, chlorogenic, and 3‐coumaroylquinic acids (hydroxycinnamic acids), as well as quercetin and kaempferol glycosides (flavonols) (Levaj et al., 2010). It also contains high levels of β‐carotene and small amounts of lutein and zeaxanthin. It is also a good source of vitamins C, B, A, E, K and minerals such as Ca, P, K, Mg (Ma & Lin, 2010; Önem, 2017; Rao & Rao, 2007). Sour cherries are generally not consumed fresh due to their high acidity and low content of simple sugars. This is the main reason for their industrial processing into juice, jam, frozen, or dried fruits (Blando & Oomah, 2019). It is thought that consumption of sour cherries can be increased by processing them into different products. One of these products is tarhana.

Tarhana is a popular cereal‐based fermented food product in Turkey. It is basically prepared with wheat flour, yogurt, yeast, different vegetables (such as onion, tomato, red pepper), and spices. It is traditionally consumed as soup by diluting it in boiling water (Certel et al., 2007; Dadalı, 2023; Koç & Özçıra, 2019). This fermented product is called by different names in different countries (Ertop et al., 2019). Tarhana is usually produced in Anatolia, Middle East, and Balkan countries, and it is known as “trahana” in Albania, “tarhana” in Bosnia‐Herzegovina, “trahana” or “tarhana” in Bulgaria, “trahanas, or zamplaricos” in Greece, “atole” in Scotland, “tarana” in Macedonia, “tarhonya” in Hungaria, “tahonya or thanu”, “talkuna” in Finland, “kishk” in Syria, Jordan, Palestine, Lebanon and Egypt, “kışk or kushuk” in Iran and Iraq, and “göce” in Turkestan (Tangüler & Erten, 2009).

Tarhana production methods (fermentation time, drying time, and temperature) and tarhana formulations (ingredients and their amounts) may vary from region to region (Kömürcü and Bilgiçli, 2022). Tarhana is basically produced by kneading the spices and pressed pasteurized vegetables with flour and yogurt and then subjecting the mixture to lactic and alcoholic fermentation for 4–5 days at room temperature. The mixture is then dried and powdered. This powder is then usually used in soup making (Bilgin et al., 2022).

Tarhana, known as a food rich in protein, vitamins, and minerals, is important in terms of balanced nutrition in public health (Atar & Özsisli, 2022). Tarhana contains various organic acids, free amino acids, and B group vitamins (thiamine, riboflavin, niacin, and vitamin B_12_). Tarhana also contains minerals obtained from vegetables, dairy products, and cereals used in the production. Moreover, it contains metabolites formed during fermentation (Tanguler & Tatlısoy, 2022). Tarhana is an important source of quercetin, a dietary flavonoid with potential in the prevention of chronic diseases, including cardiovascular and neurodegenerative diseases and cancer (Ersoy Omeroglu et al., 2023). Tarhana has an acidic and sour taste with a strong aroma, and it can be diluted to make soup. It is a good source of protein, vitamins, and minerals. For this reason, it is widely used in the nutrition of children and the elderly, and it gains importance in meeting their nutritional needs (Celik et al., 2005; Daglioǧlu, 2000; Kiliç Keskin et al., 2022).

In tarhana production, the usage of different fruits and fruit parts including tomato seeds (Işik & Yapar, 2017), grape seed extract (Akan & Ocak, 2019), pomegranate seeds (Erol & Özdestan Ocak, 2020), quince (Gökmen, 2009), cranberry (Işık et al., 2014; Karademir & Yalçın, 2019), carob (Işık Erol, 2010), blackcurrant pulp (Temiz & Tarakçı, 2017), citrus (Magala et al., 2015), raspberry, blackberry, black mulberry, rosehip, and blueberry (İstek et al., 2021; Şemşimoğlu, 2019) was reported in literature.

However, there is no study in literature in which sour cherry puree was used instead of tomato and pepper pulp and yogurt in the traditional formulation of tarhana, and its nutritional value and physicochemical properties were also examined for the first time. This study was carried out in order to contribute to the literature on the effect of sour cherry puree on nutritional and sensorial properties of tarhana, a fermented product frequently consumed in many countries, and to contribute to product diversity in order to consume more tarhana.

## 2 MATERIALS AND METHODS

### 2.1 Materials

Wheat flour (Söke Un, Aydın), commercial set‐type yogurt (at least 3.8% fat) made of cow's milk (Sütaş, Bursa), dried mint, onion, tomato, red pepper, sour cherry, and salt (Horoz tuz, Denizli) used in tarhana production were supplied from various markets in Denizli province. The sour cherries were supplied frozen and left at room temperature to thaw for 1 h before use. Onion, tomatoes, and red pepper were procured afresh.

### 2.2 Methods

#### 2.2.1 Tarhana production

The formulations given in Table 1 were used in tarhana production. The formulations were determined by preliminary experiments in the laboratory. Tarhana was produced using the method of Göncü and Celik (2020). Initially, sour cherries, onion, tomato, and red pepper to be used in tarhana production were washed. All ingredients except sour cherries were boiled separately in a stainless steel pot and left to cool at room temperature for 10 min. After that, the seeds and stems of tomatoes, peppers, onions, and sour cherries were removed, and they were finely ground into a puree using a grinder (Arzum, Turkey), which operates at a power of 2000 W (for 45 s at medium speed). The purees were mixed with yogurt, dried mint, half of the salt, and flour in the amounts given in Table 1, for 3 min at 50 rpm (Kenwood, UK), and the resulting tarhana dough was left for fermentation at 30°C for 5 days. The remaining salt was added to the fermented samples and mixed. The reason for using half of the salt later is to prevent any adverse effects on the development of lactic acid bacteria.

**TABLE 1:** Tarhana formulations.

Ingredients (%)	CT0	CT1	CT2	CT3
Flour	50.80	50.80	50.80	50.80
Yogurt	23.40	23.40	23.40	0.00
Tomato puree	11.70	0.00	5.85	11.70
Red pepper puree	7.80	0.00	3.90	7.80
Sour cherry puree	0.00	19.50	9.75	23.40
Onion puree	3.10	3.10	3.10	3.10
Dried mint	1.60	1.60	1.60	1.60
Salt	1.60	1.60	1.60	1.60

*Note*: CT0: control tarhana without sour cherry puree; CT1: tarhana produced by using sour cherry puree instead of tomato and pepper puree; CT2: tarhana produced by replacing half of the tomato and pepper purees with sour cherry puree; CT3: tarhana produced by using sour cherry puree instead of yogurt.

The dough was divided into 5–6 g pieces in trays and dried under room conditions (traditional) until the moisture content dropped to 10%. The dried tarhana samples were ground in a blender (Waring, USA) until they were smaller than 400 μm and placed in glass jars and stored in the dark and at room temperature until analyzed. Tarhana production was carried out in two replicates, and analyses were carried out in two parallel experiments.

#### 2.2.2 Physicochemical analyses

Moisture (method 934.01), ash (method 942.05), fat (method 954.02), and protein (method 988.05) analyses were performed according to AOAC (2005). Water activity values (*a*_w_) of the tarhana samples and sour cherry were determined by water activity meter (FAST Lab, GBX, Ireland) by modifying the method of Demiray (2015). Color values (*L*, *a*, and *b*) were determined using Hunter‐Lab Mini Scan XE color meter (Reston, VA, USA) (Hunterlab, 1995). Using the *L*, *a*, and *b* color values, the total color change (∆*E*) relative to the control tarhana sample was calculated using Equation 1:

(1) $$\text{∆}E={\left[{\left(L0–L1\right)}^{2}+{\left(a0–a1\right)}^{2}+{\left(b0–b1\right)}^{2}\right]}^{1/2}$$

where *L*0, *a*0, and *b*0 are the color values of the control tarhana sample; *L*1, *a*1, and *b*1 are the color values of the samples compared with the control (Karaoğlu & Bedir, 2020). For the determination of pH value of the tarhana samples and sour cherry, 5 g tarhana sample and sour cherry were mixed with 100 mL distilled water in a homogenizer (IKA‐T25, IKA‐Werke, Germany) for 3 min and then filtered through coarse filter paper, and then pH value was measured (Ibanoglu et al., 1995).

#### 2.2.3 Bioactive properties

##### Total phenolic content

For the determination of phenolic content of the samples, first extraction procedure was applied. For the extraction, initially methanol (70%, v/v) was added to the tarhana samples and sour cherry at a ratio of 1:10 (w/v), and then the samples were homogenized in a homogenizer (IKA‐T25, IKA‐Werke, Germany) and kept in an ultrasonic water bath (Elma E 60 H) for 10 min at room temperature. They were then mixed with a mechanical shaker (WiseShake SHO‐1D) for 15 min. They were centrifuged at 26,000 *g* for 20 min at 4°C (Hettich, Universal 30 RF, UK). Supernatants were transferred to amber bottles. The precipitate in the centrifuge tubes was subjected to the extraction process once more. Analysis was carried out according to the Folin–Ciocalteu (FC) method (Singleton et al., 1999). A calibration curve was obtained using gallic acid solutions. For the analysis, 5 mL of 1:10 (v/v) FC solution and 4 mL of 75 g/L Na_2_CO_3_ solution were added to 1 mL of sample extract. The resulting solutions were kept at room conditions and in a dark atmosphere for 2 h. The absorbance values were then read at 760 nm using a spectrophotometer (PG‐80 UV–Vis Spectrometer, PG Instruments, UK). Total phenolic content in 1 g of tarhana sample was expressed as milligrams of gallic acid equivalents (GAE).

##### Antioxidant activity

The antioxidant activity of the samples was measured according to the 2.2‐diphenyl‐1‐picrylhydrazyl (DPPH) method (Thaipong et al., 2006). The extraction process was carried out as described above for the total phenolic content analysis. Calibration curves were obtained with Trolox solutions in the range of 10–50 μM. The stock solution was obtained by diluting 24 mg DPPH with methanol to 100 mL and kept at −20°C. The working solution was prepared by adding 45 mL of methanol to 10 mL of the stock solution. The absorbance of the working solution was adjusted to 1.1 ± 0.02 at 515 nm wavelength in a spectrophotometer. For the analysis, 150 μL tarhana extract was mixed with 2850 μL DPPH working solution and kept in the dark and at room temperature for 1 hour. Absorbance values were then measured at 515 nm using spectrophotometer. The results were reported as μmol Trolox equivalent (TE)/100 g sample.

#### 2.2.4 Toxicological properties

The formation of 5‐hydroxymethylfurfural (HMF) was investigated to determine the toxicological properties of the tarhana samples and sour cherry. HMF analysis was performed using Agilent 1100 series high‐performance liquid chromatography (HPLC) system equipped with a DAD detector (285 nm). Initially, 6.9 g of sample was weighed and diluted to 50 mL with HPLC‐grade water. The samples were then filtered through a 0.45 μm membrane filter to remove impurities, and 20 μL of the sample was injected into the HPLC system. HMF content of the samples was determined using the calibration curve prepared with different concentrations of HMF standard. The mobile phase was prepared with 80% water and 20% methanol at a flow rate of 1 mL/min. Chromatographic separation was done using a C18 column (particle diameter: 3 μm, L × ID; 150 × 4.6) (Koç, 2015).

#### 2.2.5 Functional properties of tarhana

For water and oil absorption capacity measurements, 5 g of sample was thoroughly mixed with either 25 mL of distilled water or sunflower oil in 50 mL centrifuge tubes. The dispersions were mixed at 15‐min intervals over a period of 60 min and then centrifuged at 4000 rpm for 20 min. Water and oil absorption capacity values were expressed as g of water or oil absorbed per g of tarhana. In order to determine the foam‐forming capacity, 10 g of the sample was dispersed in distilled water and stirred for 20 min. The mixture was centrifuged at 4000 rpm for 20 min. The resulting supernatant was filtered through Whatman No. 1 filter paper, transferred to a mixer (Waring, USA), and mixed at high speed for 2 min. The solution was slowly poured into a cylinder, and the volume of foam was recorded after 10 s. The foam‐forming capacity was expressed as the volume (mL) of gas included per mL of solution (Hayta et al., 2002).

#### 2.2.6 Fatty acid composition

The oil phases of the tarhana samples and sour cherry were first extracted using a rapid oil extraction instrument (Velp Scientifica, Italy). *n*‐hexane was used as solvent in the extraction process. After extraction, methyl esters of the oil samples were prepared according to the IUPAC (1987) method. Chromatographic separation of fatty acid methyl esters was carried out using HP‐FFAP column (30 m × 0.25 mm i.d; 0.25 μm film thickness, J&W 19091F‐433, Agilent Technologies) on a gas chromatograph (Agilent 7697A, Agilent Technologies, CA, USA) with FID detector. The detector temperature was 260°C. Nitrogen was used as carrier gas at a flow rate of 3 mL/min. The oven temperature was initially set at 100°C and increased by 10°C/min to 240°C. The injection port temperature was 225°C. The injection volume was 2 μL. The split ratio was set to 100:1. Fatty acid methyl esters were identified using FAME mix (Supelco 37 component FAME mix, Sigma‐Aldrich Chemie GmbH, Taufkirchen, Germany), and results were expressed as percent methyl esters.

#### 2.2.7 Amino acid composition

Amino acid composition of the tarhana samples was determined using LC–MS/MS system. For this purpose, amino acid analysis kit (Jasem LC–MS/MS) was used. The concentrations of amino acids were determined using multiple reaction monitoring (MRM) mode based on electrospray ionization (ESI). 4 mL of reagent 2 was added to 0.5 g of tarhana sample, and the sample was hydrolyzed at 110°C for 24 h. The hydrolyzate was centrifuged at 4000 rpm for 5 min at room temperature. 100 μL of the supernatant was diluted to 1 mL with distilled water. The dilution process was repeated. Kit sample preparations were prepared as follows: To 50 μL of hydrolyzate, 50 μL of stable isotope‐labeled internal standard mixture and 700 μL of reagent‐1 were added, respectively. It was then vortexed for 5 s. 3 μL of the prepared sample was injected into Agilent 1260 Infinity HPLC system (Agilent Technologies, Santa Clara, USA) equipped with a Jasem amino acid analytical column. Operation temperature was 30°C. Chromatographic separation was completed in 7.5 min with gradient mobile phase at a flow rate of 0.7 mL/min. Mass spectrometric detection (Mass detector parameters: gas temperature 150°C, gas flow 10 L/min, nebulizer pressure 40 psi and capillary voltage +2000 v) was performed with an ESI‐equipped Agilent 6460 tandem mass spectrometer (Agilent Technologies) in positive ionization mode (Bilgin et al., 2018).

#### 2.2.8 Mineral matter composition

For the determination of mineral matter composition, first 6 mL of HNO_3_ and 2 mL of H_2_O_2_ were added to 0.5 g of the tarhana sample and sour cherry which was previously dried and ground in an oven at 70°C. The mixture was allowed to stand for 30 min and then subjected to wet digestion in a microwave oven. The digested samples were filtered through filter paper, and mineral matter composition was determined using an inductively coupled plasma optical emission spectrometer (ICP‐OES, Perkin Elmer, Optima 2100 DV, Massachusetts, USA). Among mineral matters, the amounts of P (%), K (%), Ca (%), Mg (%), Fe (ppm), Cu (ppm), Mn (ppm), Zn (ppm), and B (ppm) were determined (Göncü & Celik, 2020).

#### 2.2.9 Scanning electron microscopy

The surface morphologies of tarhanas were investigated using scanning electron microscopy (SEM). Prior to analysis, the tarhana samples were adhered to a carbon plate for conductivity and coated with a thin layer of gold/palladium (80:20/w:w) mixture (Quorum, Q150R ES, UK) at room temperature. The images of the samples were captured using a SEM instrument (ZEISS, SUPRA 40VP, Germany) at 5 kV. The images were obtained at a magnification of 4000× (Göncü & Celik, 2020).

#### 2.2.10 Sensory analysis

Tarhana soup was prepared using the formulation of Isik and Yapar (2014). According to this formulation, 4.5% tarhana powder, 88.3% water, 4.5% corn oil, 2.2% tomato paste, and 0.5% salt were used for the preparation of the soup. The panel consisted of 28 individuals, comprising 19 females and 9 males. Panelists included faculty members, students, and administrative staff from the Department of Food Engineering of Pamukkale University and Çine Vocational School of Aydın Adnan Menderes University. The panelists evaluated the soups on a hedonic scale ranging from 1 to 7 in terms of color, aroma, taste, consistency, and overall acceptance.

#### 2.2.11 Statistical analysis

The data were analyzed using “Minitab 16 Statistical Software”. ANOVA (One‐way analysis of variance) and Tukey's test (Multiple comparison test) were employed to detect significant differences (*α* = 0.05). Additionally, Principal Component Analysis (PCA) was applied to the samples in sensory evaluation, and XLstat (2022) statistical software package was used for the statistical evaluation of the data.

## 3 RESULTS AND DISCUSSION

The general composition of sour cherry samples is presented in Table 2. The moisture, protein, fat, and ash content of the sour cherry samples were 84.68%, 1.13%, 1.36%, and 0.49%, respectively. Additionally, the water activity and pH values were detected as 0.94 and 3.91, accordingly. The results were in accordance with the previously reported literature. Mahmood et al. (2013) reported that the moisture content of the sour cherry samples from different maturity stages was between 74.84% and 81.57%, while ash content was between 4.21 and 5.36 and crude protein content was 4.23%–5.91%. Toliba (2018) stated that fresh Indian cherry fruit pulp contained 78.97% moisture, 0.98% crude protein, 1.03% fat, and 0.56% ash. The color (*L*, *a*, and *b*) values of the sour cherry samples were detected as 11.06, 32.40, and 13.67, respectively. In the study of Karakurt et al. (2011), it was shown that the external brightness (*L*), redness (*a*), and yellowness (*b*) values of the sour cherry were 24.71, 16.03, and 3.77, while *L*, *a*, and *b* values of the internal part of the sour cherry were 20.78, 21.78, and 7.68, respectively.

**TABLE 2:** General composition of sour cherry.

Composition	Content	Fatty acid (%)	Content	Mineral matter	Content
Moisture (%)	84.68 ± 0.04	Palmitic acid (C16:0)	36.33 ± 10.84	P (%)	0.04 ± 0.01
Protein (%)	1.13 ± 0.05	Palmitoleic acid (C16:1)	4.00 ± 0.82	K (%)	0.23 ± 0.01
Fat (%)	1.36 ± 0.15	Heptadecenoic acid (C17:1)	2.10 ± 2.78	Ca (%)	0.30 ± 0.01
Ash (%)	0.49 ± 0.07	Stearic acid (C18:0)	14.20 ± 0.94	Mg (%)	0.02 ± 0.01
*a*_w_	0.94 ± 0.01	Oleic acid (C18:1)	37.11 ± 0.98	Fe (ppm)	6.70 ± 0.04
pH	3.91 ± 0.04	Linoleic acid (C18:2)	0.30 ± 0.08	Cu (ppm)	0.94 ± 0.02
*L*	11.06 ± 1.00	Linolenic acid (C18:3)	3.41 ± 3.82	Mn (ppm)	0.91 ± 0.02
*a*	32.40 ± 0.36			Zn (ppm)	2.12 ± 0.06
*b*	13.67 ± 0.76			B (ppm)	8.00 ± 0.01
Total phenolic content (mg GAE/g)	12.70 ± 0.99				
Antioxidant activity (μmol TE/100 g)	2.76 ± 0.37				
HMF content (ppm)	6.03 ± 0.01				

Sour cherry samples had a total phenolic content of 12.70 mg GAE/g, while their antioxidant activity was detected as 2.76 μmol TE/100 g. Wojdyło et al. (2014) reported that the content of total polyphenols ranged from 2982.51 (“Wisok” cultivar) to 1539.43 mg/100 g dry weight (“Erdi Nagygyϋmϋscu” cultivar). Their antioxidant activity ranged between 3.72 and 18.40 s mmol Trolox/100 g dm. The fluctuation in phenolic compound levels in fruits is influenced by multiple factors, including harvest maturity, environmental factors during growth, etc. Nonetheless, the cultivation of fruits with abundant nutritional elements and robust antioxidant properties is predominantly determined by genetic factors rather than climatic conditions and farming methods (Wojdyło et al., 2014; Zadernowski et al., 2005).

The dominating fatty acid of the sour cherry samples was oleic acid (37.11%) which was followed by palmitic (36.33%) and stearic acids (14.20%), respectively. Among monounsaturated fatty acids, the existence of palmitoleic (4.00%) and heptadecenoic acids (2.10%) were detected. Additionally, linoleic (0.30%) and linolenic acids (3.41%) were the polyunsaturated fatty acids of the sour cherry samples. Górnaś et al. (2016) reported that of the sour cherry kernel oils were oleic, linoleic, palmitic, and linolenic acids. Straccia et al. (2012), on the other side, reported that cherry seed oils extracted by supercritical fluid extraction contained 35.43% saturated fatty acids in addition to unsaturated (19.93%) and polyunsaturated (36.04%) fatty acids. Moreover, Ersoy et al. (2018) stated that the major fatty acid of six Cornelian cherry genotypes selected from Anatolia was linoleic acid, which was followed by oleic and palmitic acids, respectively.

Minerals are part of a vital nutrient category that is ingested in smaller amounts compared to macronutrients, yet they hold equal significance for human health. These inorganic nutrients play various roles in the body, as extensively examined by Soetan et al. (2010). In the current study, it was demonstrated that the major mineral matter of the sour cherry was B (8.00 ppm), which was followed by Fe (6.70 ppm) and Zn (2.12 ppm). In addition to major minerals, the existence of P, K, Ca, Mg, Cu, and Mn were detected. Teslić et al. (2023) stated that the most abundant mineral in sour cherry waste was Na (863.65 mg/kg), while Cu (0.31) and Mn (0.41 mg/kg) were detected in lower concentrations. On the other side, Zan et al. (2022) reported that mahaleb sour cherry contained K (433.59 mg/100 g FW), Mg (56.90 mg/100 g FW), and Ca (44.60 mg/100 g FW) as the major minerals. The fluctuations in mineral contents may be due to development stages, rootstocks, cultivation varieties, thinning and pruning methods, weather conditions in the growing season, and other factors as was previously stated by Ross et al. (2020).

Physicochemical properties of tarhana samples are given in Table 3. Moisture content, water activity (*a*_w_), ash content, and pH values were found to be statistically similar (*p* > .05). Sour cherry puree had no effect on these values. Ersoy Omeroglu et al. (2023) collected 96 tarhana samples and found moisture contents between 4.39% and 18.66% in their study. The results of the current study are within this range. According to Turkish standards (Anonymous, 1981), tarhana should have a moisture content of no more than 10%. The results were found to be compatible with Turkish standards.

**TABLE 3:** Physicochemical properties of tarhana samples.

Tarhana properties	CT0	CT1	CT2	CT3
Physicochemical properties^1^				
Moisture (%)	9.19 ± 0.14	9.51 ± 0.74	9.62 ± 0.57	9.17 ± 0.39
Protein (%)	14.84 ± 0.12^a^	12.14 ± 0.18^c^	13.23 ± 0.16^b^	10.71 ± 0.13^d^
Fat (%)	3.26 ± 0.01^a^	3.91 ± 0.70^a^	3.39 ± 0.01^a^	0.38 ± 0.01^b^
Ash (%)	1.28 ± 0.26	1.60 ± 0.13	1.48 ± 0.42	1.38 ± 0.07
*a*_w_	0.49 ± 0.01	0.50 ± 0.01	0.51 ± 0.01	0.49 ± 0.01
pH	4.04 ± 0.01	4.17 ± 0.06	4.11 ± 0.06	4.34 ± 0.14
Color properties				
*L*	48.18 ± 0.55^a^	43.91 ± 0.58^b^	47.67 ± 0.08^a^	43.46 ± 0.43^b^
*a*	9.62 ± 0.04^a^	4.26 ± 0.23^c^	5.87 ± 0.30^b^	5.93 ± 0.27^b^
*b*	16.08 ± 0.21^a^	7.45 ± 0.04^d^	11.03 ± 0.30^b^	8.89 ± 0.05^c^
Δ*E*		11.04 ± 0.48^a^	5.19 ± 0.92^b^	9.36 ± 0.10^a^
Bioactive properties^1^				
Total phenolic content (mg GAE/g)	1.70 ± 0.04^c^	1.97 ± 0.24^b^	1.76 ± 0.57^c^	2.38 ± 0.18^a^
Antioxidant activity (μmol TE/100 g)	8.53 ± 0.18^c^	9.78 ± 0.23^b^	9.66 ± 0.50^b^	10.35 ± 0.27^a^
Toxicological property^1^				
HMF content (ppm)	6.36 ± 0.15	6.51 ± 0.35	6.19 ± 0.02	7.19 ± 0.32
Functional properties				
Water absorption capacity (mL/g)	1.53 ± 0.09	1.52 ± 0.11	1.52 ± 0.10	1.56 ± 0.02
Oil absorption capacity (mL/g)	1.79 ± 0.04	1.76 ± 0.13	1.79 ± 0.14	1.84 ± 0.02
Foam‐forming capacity (mL/mL)	1.07 ± 0.07^a^	0.82 ± 0.07^b^	0.99 ± 0.04^ab^	0.97 ± 0.07^ab^

*Note*: The values displayed with different letters in the same line are statistically different from each other (*p* < .05). CT0: control tarhana without sour cherry puree; CT1: tarhana produced by using sour cherry puree instead of tomato and pepper puree; CT2: tarhana produced by replacing half of the tomato and pepper purees with sour cherry puree; CT3: tarhana produced by using sour cherry puree instead of yogurt.

^1^ The results are presented based on dry matter basis.

The highest protein content was observed for CT0 sample, and the lowest protein and fat contents were observed for CT3 tarhana sample. The reason for this situation is that unlike the others, full‐fat yogurt was not used in the production of CT3 sample. Similarly, tarhana samples produced with boza instead of yogurt (Göncü, 2020), protein, and fat amounts were found to be lower than tarhana samples produced with yogurt. In the study of Ertugay et al. (2000), protein was reported as 14.2%, ash 1.5%, and fat 2.1%. When the control tarhanas were compared, CT0 was found to be richer in protein and fat but lower in ash. In the review study of Bilgin et al. (2022), the analysis results of 15 different tarhanas were presented as follows: moisture 4.8%–11.7%, protein 5.3%–17.3%, and fat 2.3%–6.2%. The results obtained are within these ranges. In contrast to these results, the results obtained in the study of Atar and Özsisli (2022) (ash 2.7%, protein 16.9%, fat 12%, pH 4.9) were lower than the findings. Changes in physicochemical properties were observed in parallel with changes in tarhana formulation. As shown in Table 2, low protein and fat content of the sour cherry could be reduced the tarhana values.

When the color characteristics of tarhana samples were considered, it was determined that CT0 and CT2 were lighter. As the amount of the sour cherry puree increased, the color of the tarhana became darker. Redness (a) and yellowness (b) values were highest in CT0. This may be due to the fact that tomato and pepper purees contain color pigments with higher redness and yellowness values than sour cherry puree. These results were also determined for total color changes (Δ*E*). The CT1 sample has the highest Δ*E* value. This showed that the sample with the most different color from the control sample is CT1. Dadalı (2023) determined the color values of tarhana obtained with artichoke leaves as *L*: 54.39–64.49, *a*: 5.40–19.22, *b*: 28.54–44.89, and Δ*E*: 19.66–23.68. Kiliç Keskin et al. (2022) found the *L*, *a* and *b* color values of tarhanas obtained with legumes in the following range, respectively: 68.12–77.71, 8.80–11.78, and 31.59–37.71. Karademir and Yalçın (2019) reported that *L* value in cranberry tarhana was 67.62–76.93, *a* value was 7.89–19.42, and *b* value was 6.70–12.86. The *L* values of the tarhana obtained with sour cherry puree were lower than the values reported in this study, the *a* value was within the given ranges except for the CT1 sample, the *b* value was within the given ranges, and the Δ*E* value was much lower. The differences in color values are due to the different raw materials used in tarhana production. It is thought that the color changes differ due to the rich anthocyanin, lycopene, and other color pigments contained in the pepper, tomato, and sour cherry purees in the formulation. The *a* and *b* values decreased in tarhana produced using cranberries (Işık et al., 2014), as in sour cherries.

When the bioactive properties of the tarhana samples were analyzed (Table 3), it was observed that the lowest total phenolic content and antioxidant activity values belonged to the CT0 sample. When sour cherry puree, tomato, and pepper purees were used instead of yogurt, it was found that there was an increase in antioxidant activity and total phenolic matter content. In the study of Cagindi et al. (2016), 27 different local and commercially sold tarhana samples in Turkey were analyzed, and total phenolic contents were determined. The results were given in the range of 0.055–4.26 mgGAE/g. The results of the present study are within this range. In the study of Tanguler and Tatlısoy (2022), the total phenolic content (1.43 mgGAE/g) was lower than the result obtained in the present study. Işik and Yapar (2017) reported the total phenolic content and antioxidant activity of control tarhana samples as approximately 2.00 mg GAE/g and 11.00 μmol TE/100 g, respectively. When the results were examined, total phenolic content and antioxidant activity values were lower in the present study. Likewise, in the study of Dadalı (2023), it was determined that tarhana samples with artichoke leaves fortification contained much higher total phenolic content (2.88–3.62 mg GAE/g) and antioxidant activity value (3.07–3.86 μmol TE/g) than tarhana samples with sour cherry puree. It was observed that there is an increase in total phenolic component and antioxidant activity in tarhanas enriched with fruit. This is due to the fact that fruits contain rich phenolic components, and these components have antioxidant capacity. Increasing antioxidant activity provides many health benefits for individuals. In addition, phenolic components reflect their unique aromas to the foods they are added to (Tarakci et al., 2013). 5‐Hydroxymethylfurfural (HMF) contents of tarhana samples were found in the range of 6.19–7.19 ppm. There was no significant (*p* > .05) difference between the samples. In the literature, there were no studies investigating HMF content of tarhana. The estimated daily intake dose of HMF is up to 30–150 mg per person, which is equal to an estimated 2.5 mg/kg body weight per person (Batu et al., 2014). When the HMF amounts of tarhana samples are examined, they can be considered safe by staying below the intake dose in adult individuals.

When the tarhanas were analyzed in terms of their functional properties, there was no statistical difference (*p* > .05) in terms of water and oil absorption capacities. However, the highest foam‐forming capacity was found for CT0 sample. Foaming capacity decreased as the use of sour cherry puree increased. Hayta et al. (2002) reported the foaming capacity of tarhana as 0.11–0.65 mL/mL, water absorption capacity as 0.45–2.28 mL/g, and oil absorption capacity as 0.62–0.76 mL/g; Kömürcü and Bilgiçli (2022) reported the foaming capacity as 1.13–1.20 mL/mL, water absorption capacity as 1.15–1.19 mL/g, and oil absorption capacity as 1.23–1.66 mL/g. Bilgiçli (2009) reported the foaming capacity of buckwheat‐added tarhana samples between 0.75 and 1.91 mL/mL. While the foaming capacity and water absorption capacity were found to be within the ranges given in the studies, the oil absorption capacity was determined to be higher. The wide range of functional properties of tarhanas in the literature may be due to the differences in formulations and due to the properties of proteins found in raw materials. It was thought that the reason for the decrease in foaming capacity, when sour cherry is included in the tarhana formulation, is related to the amount and types of protein in the samples. Tarakci et al. (2013) reported that various proteins from yogurt and wheat may change gas absorption, and moreover protein diversity affects the foaming capacity.

The amino acid composition of the tarhana samples is given in Table 4. CT3 was found to be the poorest formulation in terms of amino acid diversity. The reason for this is the use of sour cherry puree instead of yogurt in the formulation of CT3. Alanine, arginine, aspartic acid, glutamic acid, histidine, leucine, phenylalanine, phenylalanine, proline, and threonine amino acids were mostly detected in yogurt samples containing sour cherry puree. Additionally, yogurt and sour cherry puree samples were the richest samples in terms of essential amino acids. Cystine and glycine were similar (*p* > .05) in all samples. Similar to CT3, in Göncü's study (2020), boza was used instead of yogurt in tarhana formulation, and it was determined that alanine, glutamic acid, lysine, phenylalanine, proline, serine, cystine, threonine, and valine amounts were found to be lower.

**TABLE 4:** Amino acid composition of tarhana samples^1^.

Amino acid (mg/100 g)	CT0	CT1	CT2	CT3
Alanine	535.05 ± 7.88^bc^	656.12 ± 7.04^a^	579.38 ± 8.93^b^	491.50 ± 0.15^c^
Arginine	497.69 ± 3.26^ab^	513.63 ± 10.43^a^	520.13 ± 5.05^a^	461.50 ± 17.24^b^
Aspartic acid	659.35 ± 14.08^c^	852.21 ± 8.12^a^	747.96 ± 3.34^b^	537.35 ± 9.91^d^
Cystine	281.86 ± 20.98	297.92 ± 16.05	282.00 ± 4.72	280.03 ± 6.55
Glutamic acid	4248.41 ± 3.22^c^	4677.57 ± 5.41^a^	4653.15 ± 4.72^b^	4192.94 ± 3.13^d^
Glycine	367.51 ± 63.39	449.63 ± 18.04	397.13 ± 76.13	316.88 ± 5.92
Histidine^2^	488.15 ± 6.90^b^	534.51 ± 13.04^ab^	541.60 ± 1.84^a^	404.13 ± 19.47^c^
Isoleucine^2^	434.08 ± 1.38^a^	422.69 ± 3.49^a^	443.78 ± 18.39^a^	314.55 ± 13.55^b^
Leucine^2^	1052.80 ± 18.01^b^	1179.30 ± 6.40^a^	1147.40 ± 41.40^ab^	867.60 ± 13.30^c^
Lysine^2^	798.55 ± 2.40^a^	716.86 ± 0.69^b^	796.51 ± 13.11^a^	528.68 ± 28.02^c^
Methionine^2^	231.99 ± 2.99^a^	244.76 ± 7.81^a^	244.94 ± 9.81^a^	197.16 ± 6.52^b^
Phenylalanine^2^	789.32 ± 17.81^b^	848.97 ± 9.13^a^	862.46 ± 9.69^a^	742.54 ± 3.27^c^
Proline	1742.30 ± 19.30^b^	1816.40 ± 13.40^a^	1858.90 ± 0.40^a^	1638.40 ± 0.40^c^
Serine	885.58 ± 3.32^a^	827.88 ± 3.21^c^	863.57 ± 5.98^b^	708.30 ± 6.99^d^
Threonine^2^	482.07 ± 4.55^ab^	546.59 ± 29.83^a^	539.14 ± 3.50^a^	432.80 ± 23.65^b^
Tyrosine	211.46 ± 3.32^b^	250.85 ± 15.17^a^	233.07 ± 0.36^ab^	218.13 ± 2.74^b^
Valine^2^	628.41 ± 0.46^c^	667.48 ± 0.17^b^	674.33 ± 0.12^a^	498.26 ± 0.57^d^

*Note*: The values displayed with different letters in the same line are statistically different from each other (*p* < .05). CT0: control tarhana without sour cherry puree; CT1: tarhana produced by using sour cherry puree instead of tomato and pepper puree; CT2: tarhana produced by replacing half of the tomato and pepper purees with sour cherry puree; CT3: tarhana produced by using sour cherry puree instead of yogurt.

^1^ The results are presented based on dry matter basis.

^2^ Essential amino acid.

Işik and Yapar (2017) determined the amino acid composition of tarhana enriched with tomato seeds. The addition of tomato seeds caused an increase in most of the amino acids. In the current study, the amount of amino acids increased as sour cherry puree increased in yogurt formulations. When compared to the control tarhana, histidine, lysine, and serine amino acids were higher in the present study, while other amino acids were determined in lower amounts. In the study of Daglioǧlu (2000), amino acid amounts of different tarhanas were reported. When compared with the current study, arginine, aspartic acid, threonine, glutamic acid, proline, glycine, and isoleucine were lower than the given amounts, cystine was higher, and other amino acids were within the given ranges. It is thought that the differences in amino acid amounts are due to the differences in formulations and production methods. Wu (2009) reported that amino acids are cell signaling molecules, regulators of gene expression, key precursors for the protein phosphorylation cascade and hormone synthesis, and are of great biological importance. Enriching tarhana with sour cherries, which are rich in amino acids, is important for human health.

The fatty acid composition of the tarhana samples is given in Table 5. The amount of saturated fatty acids (palmitic and stearic acids) decreased in the yogurt samples with sour cherry puree but did not change in the yogurt‐free samples (*p* > .05). The amounts of unsaturated fatty acids palmitoleic, heptadecenoic, and oleic acids decreased significantly in the yogurt‐free samples (CT3). The amounts of linoleic and linolenic acids, which are polyunsaturated fatty acids, were highest in the samples without yogurt and lowest in the control (CT0) and samples without vegetable puree (CT1). The highest amount of oleic acid was found in the tarhana samples with yogurt. The highest amount of linoleic acid was found in the samples without yogurt.

**TABLE 5:** Fatty acid composition of tarhana samples.

Fatty acid (%)	CT0	CT1	CT2	CT3
Palmitic acid (C16:0)	12.63 ± 1.22^a^	9.36 ± 0.85^b^	8.05 ± 0.03^c^	12.89 ± 0.25^a^
Palmitoleic acid (C16:1)	20.37 ± 0.08^a^	19.51 ± 2.96^a^	18.93 ± 2.44^a^	4.80 ± 0.49^b^
Heptadecenoic acid (C17:1)	1.89 ± 0.78^a^	1.37 ± 0.25^a^	0.49 ± 0.01^b^	0.41 ± 0.08^b^
Stearic acid (C18:0)	4.31 ± 0.74^a^	2.33 ± 0.34^b^	2.76 ± 1.74^ab^	2.87 ± 1.34^ab^
Oleic acid (C18:1)	39.12 ± 1.81^a^	36.00 ± 2.63^b^	32.61 ± 0.88^c^	22.02 ± 1.67^d^
Linoleic acid (C18:2)	12.43 ± 0.71^c^	11.09 ± 0.96^c^	16.11 ± 1.37^b^	33.56 ± 0.09^a^
Linolenic acid (C18:3)	14.04 ± 0.34^c^	14.73 ± 2.21^c^	17.51 ± 0.12^b^	28.91 ± 1.55^a^

*Note*: The values displayed with different letters in the same line are statistically different from each other (*p* < .05). CT0: control tarhana without sour cherry puree; CT1: tarhana produced by using sour cherry puree instead of tomato and pepper puree; CT2: tarhana produced by replacing half of the tomato and pepper purees with sour cherry puree; CT3: tarhana produced by using sour cherry puree instead of yogurt.

The results of the current study considering stearic, oleic, and linoleic acid were within the ranges given in a study (Ovando‐Martinez et al., 2014) in which the fatty acid contents of 15 tarhana samples collected from different regions of Turkey were examined, while the amounts of palmitoleic and linolenic acids were found above the given range and palmitic acid was found below the range. Similar to the results of the present study, oleic acid was among the dominant fatty acids in some studies (Isik & Yapar, 2014; Ovando‐Martinez et al., 2014), while palmitic acid was the dominant saturated fatty acid (Erbaş et al., 2006). Additionally, the dominating fatty acid of the sour cherry samples was also oleic acid (Table 2). So it can be stated that oleic acid does not generally change significantly during fermentation as was previously reported by Lee et al. (2019) who stated that oleic acid concentrations of Chungkookjang, a fermented soybean food, were not significantly altered by fermentation. Compared to raw material (Table 2), the content of dominating fatty acid, palmitic acid, tended to decrease throughout fermentation. A similar situation was also reported by Afify et al. (2012). However, when compared to raw material (Table 2), the amount of linolenic acid tended to increase. Ansorena and Astiasarán (2004) similarly reported that the content of linolenic acid may increase during fermentation.

The mineral matter composition of tarhana samples is given in Table 6. There was no significant difference (*p* > .05) between the P and Mg values of the samples. The lowest amount of Ca was found in CT3 tarhana, which was produced without yogurt. The highest amounts of K, Fe, Cu, Mn, and B values were found in CT3 sample where tomato, pepper, and sour cherry purees were used in total, while the highest Zn value was found in CT1 sample where sour cherry puree was used instead of tomato and pepper purees. The lowest Fe and B values were observed in CT0 (control) sample. Sour cherry puree caused an increase in Fe and B contents.

**TABLE 6:** Mineral matter composition of tarhana samples^1^.

Mineral matter	CT0	CT1	CT2	CT3
P (%)	0.20 ± 0.01	0.21 ± 0.01	0.20 ± 0.01	0.18 ± 0.01
K (%)	0.33 ± 0.01^c^	0.38 ± 0.01^b^	0.36 ± 0.01^b^	0.47 ± 0.01^a^
Ca (%)	0.15 ± 0.01^a^	0.14 ± 0.01^a^	0.14 ± 0.01^a^	0.08 ± 0.01^b^
Mg (%)	0.07 ± 0.01	0.07 ± 0.01	0.06 ± 0.01	0.07 ± 0.01
Fe (ppm)	35.21 ± 0.65^d^	57.70 ± 0.04^b^	50.78 ± 0.70^c^	84.54 ± 1.01^a^
Cu (ppm)	2.41 ± 0.17^ab^	2.35 ± 0.13^ab^	2.14 ± 0.01^b^	2.78 ± 0.01^a^
Mn (ppm)	9.58 ± 0.01^ab^	8.50 ± 0.45^c^	8.89 ± 0.02^bc^	10.08 ± 0.04^a^
Zn (ppm)	11.19 ± 0.11^b^	11.95 ± 0.01^a^	10.66 ± 0.07^c^	10.03 ± 0.07^d^
B (ppm)	1.67 ± 0.07^d^	3.77 ± 0.11^b^	2.73 ± 0.02^c^	4.75 ± 0.07^a^

*Note*: The values displayed with different letters in the same line are statistically different from each other (*p* < .05). CT0: control tarhana without sour cherry puree; CT1: tarhana produced by using sour cherry puree instead of tomato and pepper puree; CT2: tarhana produced by replacing half of the tomato and pepper purees with sour cherry puree; CT3: tarhana produced by using sour cherry puree instead of yogurt.

^1^ The results are presented based on dry matter basis.

Considering Table 2, it can be stated that sour cherries are rich in terms of Fe and B, and therefore there is an increase in the Fe and B values in tarhana; on the contrary, since sour cherries are poor in terms of P and Mg, they do not have a statistically significant effect on the P and Mg values of tarhana.

The results of some studies on this subject are as follows; P (%): 0.16 (Bilgiçli et al., 2006) and 0.29 (Çağlar et al., 2013); K (%): 0.33 (Işik & Yapar, 2017) and 0.53 (Bayrakçi & Bilgiçli, 2015); Ca (%): 0.03 (Aktaş et al., 2015) and 0.14 (Işik & Yapar, 2017); Mg (%): 0.04 (Bayrakçi & Bilgiçli, 2015) and 0.07 (Bilgiçli et al., 2006); Fe (ppm): 19.80 (Bilgiçli et al., 2006) and 25.20 (Bilgiçli, 2009); Cu (ppm): 1.65 (Göncü & Celik, 2020) and 6.40 (Işik & Yapar, 2017); Mn (ppm): 5.90 (Bilgiçli et al., 2006) and 9.12 (Göncü & Celik, 2020) and Zn (ppm): 9.90 (Aktaş et al., 2015) and 12.10 (Bayrakçi & Bilgiçli, 2015). When the results are compared, Fe and Mn results were found to be higher in sour cherry puree tarhanas, while no study was found which investigated the content of B. The results obtained for other mineral substances were within the ranges given in the studies. In the tarhana samples made by Göncü (2020), boza was used instead of yogurt and the lowest amount of Ca was found in these samples as in CT3. Since the mineral content of fruits provides a large portion of people's Recommended Daily Intake (RDA) value (Temiz & Tarakçı, 2017), it can be said that the use of sour cherries contributes to this by increasing some elements in tarhana.

**FIGURE 1:**
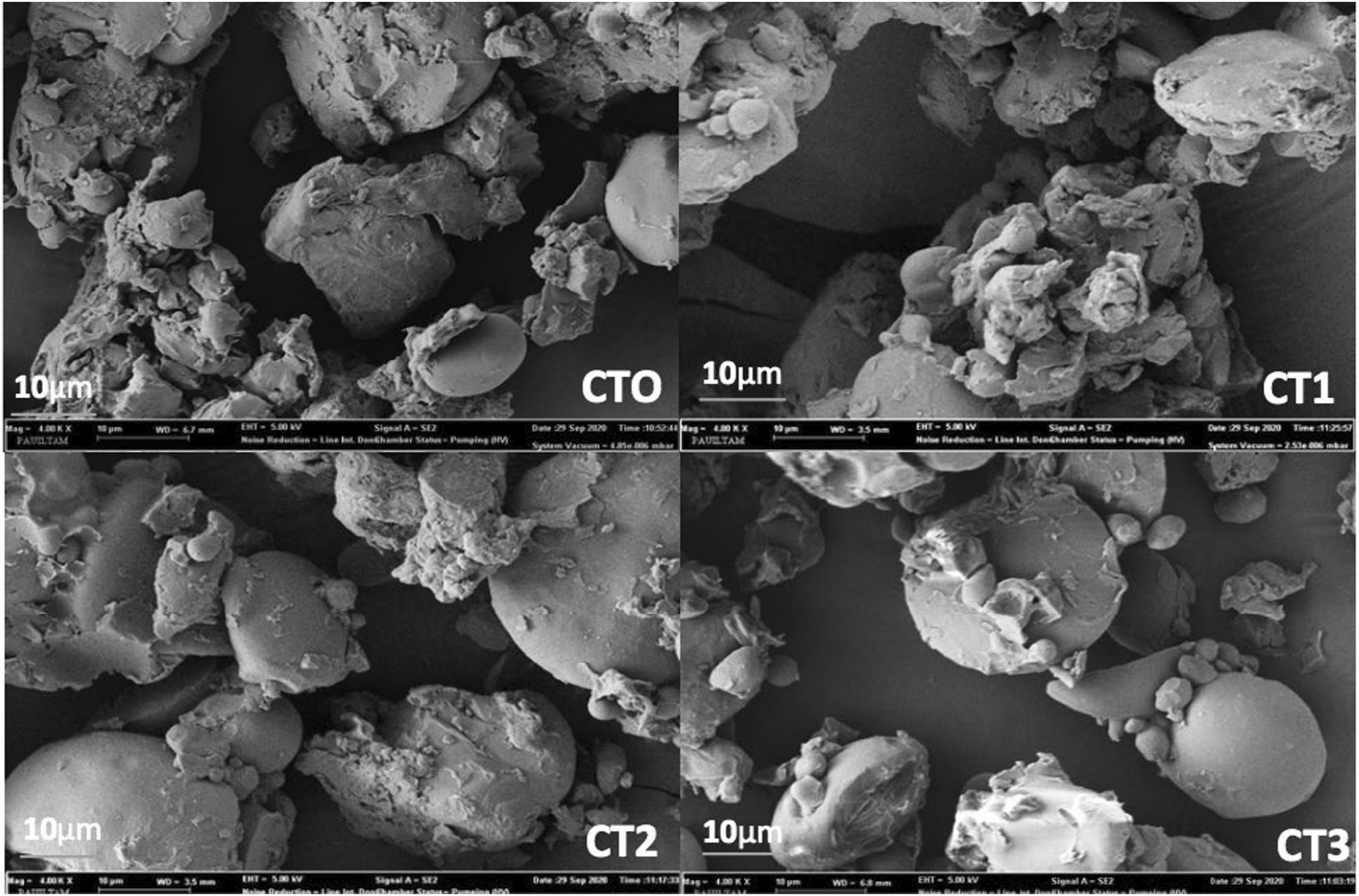
SEM images of the tarhana samples. CT0: control tarhana without sour cherry puree; CT1: tarhana produced by using sour cherry puree instead of tomato and pepper puree; CT2: tarhana produced by replacing half of the tomato and pepper purees with sour cherry puree; CT3: tarhana produced by using sour cherry puree instead of yogurt.

The surface morphology of the tarhana samples was analyzed by scanning electron microscopy (SEM). SEM images are shown in Figure 1. According to the micrographs, different particle distributions were observed. While small and irregular particle amounts were more prominent in CT0 and CT1 samples, oval structures were more prominent in CT2 and CT3 samples. Similar irregularities were also mentioned in the studies of Göncü and Celik (2020) on tarhana and Salameh et al. (2016) on kishk.

Some particles were observed to be covered with a thin layer. These were reported to be oil layers in different studies (Do et al., 2011; Salameh et al., 2016). CT3 was the sample with the least amount of these layers. Because for this sample, full‐fat yogurt was not used, which was used for other formulations, the structures seen as oval granules indicate unflavored starches. The reason for this is that the starch gelatinization temperature could not be reached during the drying of tarhana dough. Erbay (2013) stated that if the protein content on the surface of powder products is high, breakage and implosion may occur in the powder particles. Among the samples, the highest protein content was found in CT0 and the lowest in CT3 (Table 3). Therefore, breakage and migration were observed most in CT0 and least in CT3.

When the samples were analyzed in terms of their sensory properties (Table 7), it was seen that the sample in which 100% sour cherry puree was used (CT1) was least appreciated in terms of color. But the same sample was the most appreciated in terms of odor. The most liked sample in terms of flavor was CT2. According to the results, it was seen that using sour cherry puree instead of yogurt affects the consistency negatively. When tarhana samples were evaluated as general taste, it was determined that CT2 was the most liked (*p* < .05) sample together with CT0. Although CT1 got the lowest overall score (4.07), it was still above the mean value.

**TABLE 7:** Sensorial properties of tarhana samples.

Property	CT0	CT1	CT2	CT3
Color	5.64 ± 1.93^a^	3.42 ± 1.31^c^	4.67 ± 1.24^b^	4.39 ± 1.16^b^
Odor	4.78 ± 0.95^bc^	5.96 ± 0.96^a^	5.10 ± 1.03^b^	4.28 ± 1.04^c^
Taste	4.89 ± 1.06^ab^	3.60 ± 1.47^c^	5.53 ± 1.17^a^	4.60 ± 0.83^b^
Consistency	5.28 ± 0.89^a^	5.07 ± 1.24^a^	4.57 ± 1.16^ab^	4.28 ± 1.15^b^
General acceptability	5.07 ± 1.01^a^	4.07 ± 1.60^b^	5.00 ± 0.94^a^	4.53 ± 1.10^ab^

*Note*: The values displayed with different letters in the same line are statistically different from each other (*p* < .05). CT0: control tarhana without sour cherry puree; CT1: tarhana produced by using sour cherry puree instead of tomato and pepper puree; CT2: tarhana produced by replacing half of the tomato and pepper purees with sour cherry puree; CT3: tarhana produced by using sour cherry puree instead of yogurt.

**FIGURE 2:**
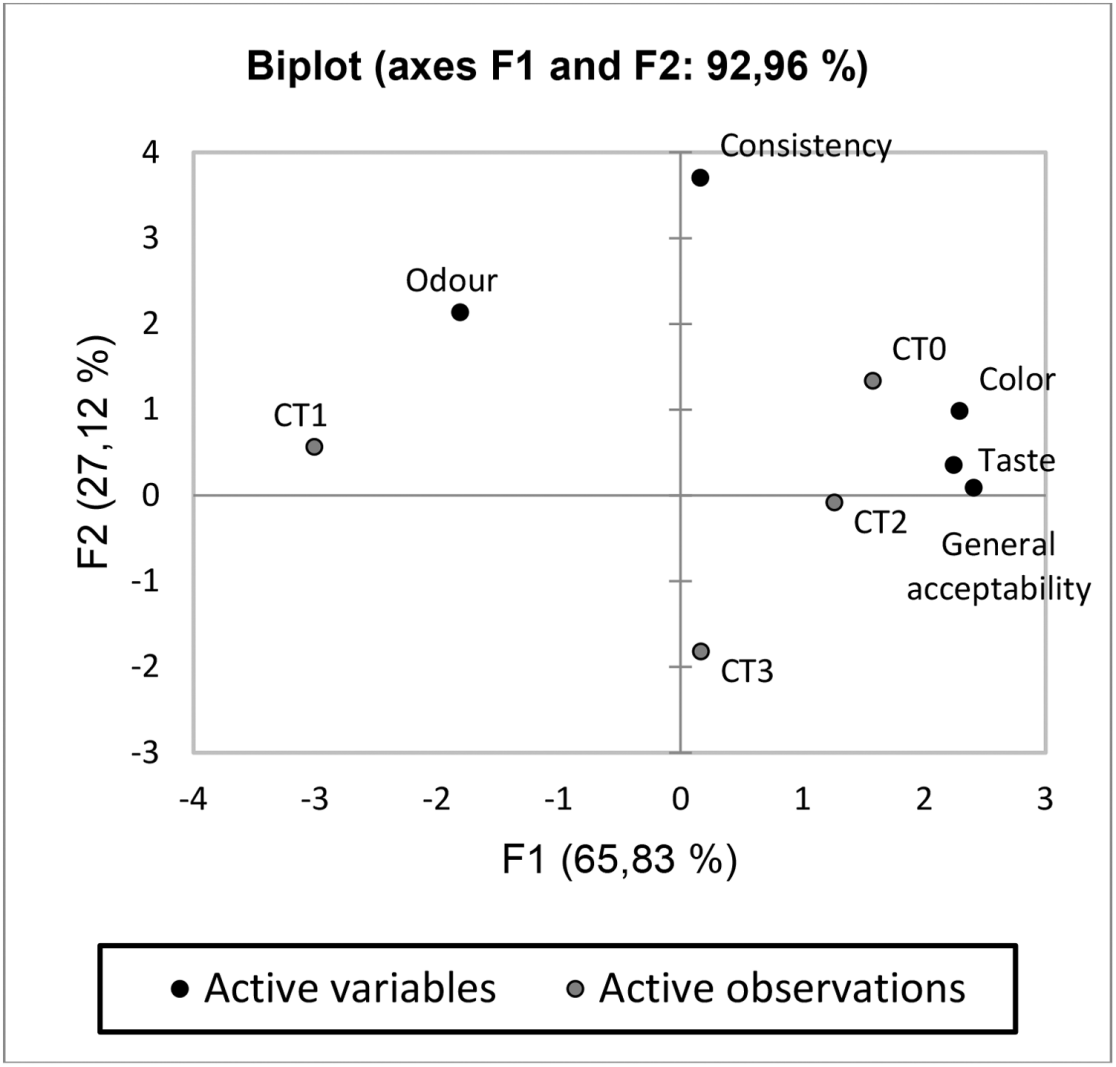
PCA biplot graph related with the sensory analyses of tarhana samples. CT0: control tarhana without sour cherry puree; CT1: tarhana produced by using sour cherry puree instead of tomato and pepper puree; CT2: tarhana produced by replacing half of the tomato and pepper purees with sour cherry puree; CT3: tarhana produced by using sour cherry puree instead of yogurt.

Principal component analysis (PCA) graphics related with the sensory analysis results of tarhana samples are shown in Figure 2. As a result of the creation of perceptual maps of tarhanas with PCA, similar flavors were grouped close to each other, while different samples were grouped separately from each other (Kitsawad & Tuntisripreecha, 2016). It also contributed to the identification of tarhana samples by characterizing them according to their similarities and differences in their sensory properties (Bal Yıldırım et al., 2021). The variance of the variables reached as a result of PCA was determined as 92.96%, 65.83% for the F1 area and 27.12% for the F2 area.

It was seen that tarhana samples in different quarters show different sensory properties. According to the graph, the color, taste, and general acceptance scores of the samples were found to be similar to each other. As can be seen from the sensory analysis results (Table 7), CT0 and CT2 samples were the most similar to each other, while CT1 and CT3 exhibited completely different characteristics.

Samples CT0 and CT2 were represented by color, flavor, and general taste, while CT1 was represented by odor. CT3 was not represented by any parameter. The reason why CT2 gets such similar scores to CT0 is the yogurt, tomato, and pepper purees found in traditional tarhana. Except for control, these three components are only included in CT2.

## 4 CONCLUSION

In this study, the possibility of using the sour cherry puree with tomato and pepper puree in the tarhana formulation in 50% and 100% ratios and substituting with 100% yogurt was investigated. Significant increases in fat, ash, amino acids, mineral matter, antioxidant activity, and total phenolic matter were observed with the use of sour cherry puree. It was determined that tarhanas were rich in oleic acid, one of the monounsaturated fatty acids. There was a decrease in *L* value. HMF content of the samples did not change significantly. When sour cherry puree substituted tarhanas were compared among themselves, CT3 was the richest in terms of mineral substances, CT1 in terms of amino acids, and CT2 in terms of protein. When the sensory analyses were considered, CT2 and CT3 received the same general acceptance scores as CT0, the control tarhana, which means that CT1 was not acceptable. According to PCA results, CT2 was the closest sample to CT0. As a result, it can be recommended that sour cherry puree can be substituted with tomato and pepper purees at a rate of 50% in tarhana production due to both nutritional enrichment of tarhana and sensory acceptability. In future studies, the effects of sour cherry puree on the flavor profile of tarhana can be investigated. The potential of different sour cherry varieties, processing and extraction types, as well as sour cherry production wastes to be used as functional raw materials in tarhana, and their effects on consumer expectations should also be investigated. These studies may contribute to the determination of new product development and marketing strategies in the tarhana industry.

## FUNDING INFORMATION

No funding was received to assist with the preparation of this manuscript.

## CONFLICT OF INTEREST STATEMENT

The authors declare that they do not have any conflict of interest.

## ETHICS STATEMENT

This study does not involve any human or animal testing.

## Data Availability

The data that support the findings of this study are available on request from the corresponding author.
